# Radiological manifestations and complications of lung and brain in Egyptian COVID-19 patients

**DOI:** 10.1186/s43055-022-00742-y

**Published:** 2022-03-21

**Authors:** Kholoud Hamdy El-Shourbagy, Lamees M. Ghith, Lina T. Hablas

**Affiliations:** Lecturer of Diagnostic Radiology, Department of Medical Imaging and Interventional Radiology, Faculty of Medicine, Tanta University, Tanta, Egypt

**Keywords:** COVID-19, Lung, Neurological manifestations, ADEM = acute disseminated encephalomyelitis, PRES = posterior reversible encephalomyelitis syndrome

## Abstract

**Background:**

Coronavirus disease COVID-19 is a viral illness, currently affecting millions of people worldwide. Pneumonia is the most common extreme presentation of COVID-19 infection, manifesting by fever, dry cough, difficulty of breathing or shortness of breath and mainly ground-glass infiltrates in radiological images. Chest computed tomography (CT) has a potential role in the diagnosis, detection of complications and prognostication of coronavirus disease COVID-19. In addition to severe respiratory manifestations, there are a wide range of neurological manifestations ranging from nonspecific symptoms to necrotizing encephalopathies and stroke. Our study aimed to review lung and neurological manifestations in recent and post-COVID-19 Egyptian patients and to be familiar with magnetic resonance imaging (MRI) findings of Neuro-COVID patients.

**Results:**

The present study included eighty COVID-19 patients with age ranged from 28 to 78 years (mean age 57.84 + 12.58 years) who were 54 males (mean age 56.64 + 12.50) and 26 females (mean age 48.65 + 14.24). All our patients were with recent or previous history of COVID-19 infection and subjected to careful history taking, thorough clinical examination, routine laboratory investigations and CT examination. The reported lung manifestations included normal lung shadows, ground-glass opacifications (GGOs), consolidations, reticulation, reticulation and GGOs (crazy paving) and fibrotic-like changes. Out of eighty COVID-19 patients, twenty showed neurological manifestations ranging from nonspecific symptoms to necrotizing encephalopathies and stroke. Patients with neurological manifestation were in addition to CT submitted to magnetic resonance imaging (MRI) as appropriate. MRI done to neuro-COVID patients showed that 8/20 (40%) had no abnormalities and 12/20 (60%) had abnormalities. The most common abnormalities are infarction, major or lacunar infarction, followed by acute disseminated encephalomyelitis (ADEM), posterior reversible encephalopathy syndrome (PRES) and meningoencephalitis.

**Conclusion:**

Old age patients, especially males, were more affected than females. Lung manifestations are common in COVID-19 patients than neurological manifestations. The presence of fibrotic changes in the lung could predict severe COVID-19 affection and bad prognosis. There might be an association between appearance of neurological manifestations and poor outcome in COVID-19 patients.

## Background

Coronavirus disease 2019 (COVID-19) is a highly contagious viral disease that may cause, in addition to lung disease, a wide range of non-respiratory complications due to involvement of organs by the virus or due to direct or indirect complications of this infection [1]. Thromboembolic complications due to abnormal coagulation presented in patients with COVID-19 infection may occur in up to 31% of COVID-19 patients in intensive care unit (ICU) [2]. COVID-19 coagulation disorders were associated with increased morbidity and mortality [3].

Pneumonia is the most common extreme presentation of COVID-19 infection, manifesting by fever, dry cough, difficulty of breathing and various radiological CT findings [4]. Chest CT is the best imaging modality that detects different parenchymal abnormalities and disease severity in COVID-19 patients [5].

The most common findings in chest CT of COVID-19 patients are GGOs, appearance of crazy-paving pattern, consolidations, thickening of interlobular septa, reticular pattern, mixed pattern, air bronchogram sign and bronchiolectasis, among others [6].

There are several mechanisms involved in COVID-19-associated CNS dysfunction, particularly activation of inflammatory and thrombotic pathways and, in a few patients, a direct viral effect on the endothelium and the parenchyma. Thrombi are a major cause of multisystem organ dysfunction, including respiratory failure as had been previously reported [7].

Types of neurologic complications in COVID-19 patients depend on state of patients. Those with mild cases present with headache or dizziness, whereas those with severe disease can also develop an encephalopathy with agitation, confusion, impaired consciousness, seizures and signs of corticospinal tract dysfunction [8]. Ischemic stroke occurring in COVID-19 might be associated with a viral-related systemic prothrombotic state [9]. Demonstration of COVID-19 in the brain or CSF has been inconsistent in acute COVID-19. There is an association between the presence of lung and neurological manifestations and mortality [10].

The aim of the present study is to review lung and neurological manifestations and complications associated with COVID-19 infection and analyze potential mechanisms of their damage and to be familiar with MRI findings of neuro-COVID patients.

## Methods

### Patients

This prospective interventional study was carried out following the approval of the Research Ethical Committee, from December 2020 to April 2021, on eighty patients; age range 28–78 years with past or present history of COVID-19 infection; collected from hospitals; ICU and out-patients clinic. The inclusion criteria included confirmed COVID-19 cases with positive PCR, and patients had history of recent contact with confirmed COVID-19-infected patients. The exclusion criteria were patients with chronic thromboembolic events in chest or brain not confirmed to be COVID-19 positive and patients with previous neurocognitive disorders. All patients were subjected to careful history taking, clinical examination, routine laboratory findings, CT examination and MRI examination as well done for patients complaining of neurological manifestations (20 patients). Informed written consent was obtained from all participants that their clinical, laboratory and radiological data had to be used in the study.

### Data collection

Demographic data (age, sex), medical history of comorbid diseases (diabetes mellitus, hypertension, chronic kidney disease, cardiac disease, cancer) and clinical data (presenting symptoms, risk factors and ICU admission) were collected from patients compiled and analyzed.

## Methods

- Chest CT was performed at time of admission for patient in acute phase. Chest CT was performed on GE 128 slice machine. The scans were obtained while the patient in supine position at end of inspiration with hands raised above head and hold his breath. Chest CT was carefully examined for each patient by three expert radiologists which interpreted the images in conjunction.
- MRI was done using a 1.5 Tesla MR scanner (GE Healthcare). MR sequence parameters were as follows: T1-weighted images parameters; T2-weighted images parameters; and diffusion weighted images.

### Image analysis

The CT severity score index was calculated for each one of the five lung lobes, by calculation of the dissemination of the chest manifestations (opacity), ground-glass opacity (GGO), consolidation, crazy-paving pattern, septal thickening and pulmonary fibrosis. The degree of consolidation and crazy-paving pattern were highly indicators for the disease severity and the disease progression/peak as formerly reported [11].

Ground-glass opacity (GGO) is an increase in the lung density, with still visible bronchial vascular bundles. On the other hand, consolidation is opacification with obscured underlying vasculature. Fibrosis was defined as parenchymal bands, irregular interfaces (bronchovascular, pleural or mediastinal), coarse reticular pattern and traction bronchiectasis [12].

To quantify the extent of pulmonary abnormalities (total lesions, GGO, consolidation, reticulation and fibrotic-like changes), a semi-quantitative CT score [13] was assigned based on the area involved in each of the five lung lobes and given a score from 1 to 5:

1. Representing less than 5% lobar involvement.
2. 5–25% lobar involvement.
3. 26–50% lobar involvement.
4. 51–75% lobar involvement.
5. 75% lobar involvement.

Then, the final score will be the summation of individual lobar scores and will be out of 25 (total score); the total lung involvement is mild ≤ 10, moderate 11–20 and severe ≥ 20.

Patients especially those having severe neurological manifestations had done MRI of the brain in addition to CT of chest (20 cases).

### Statistics

Data of the present study were described as mean ± standard deviation (SD) or number and frequency percent. All statistical operations done using SPSS 26 (IBM, IL, USA).

## Results

### Demographic and participant characteristics

**Fig. 1:**
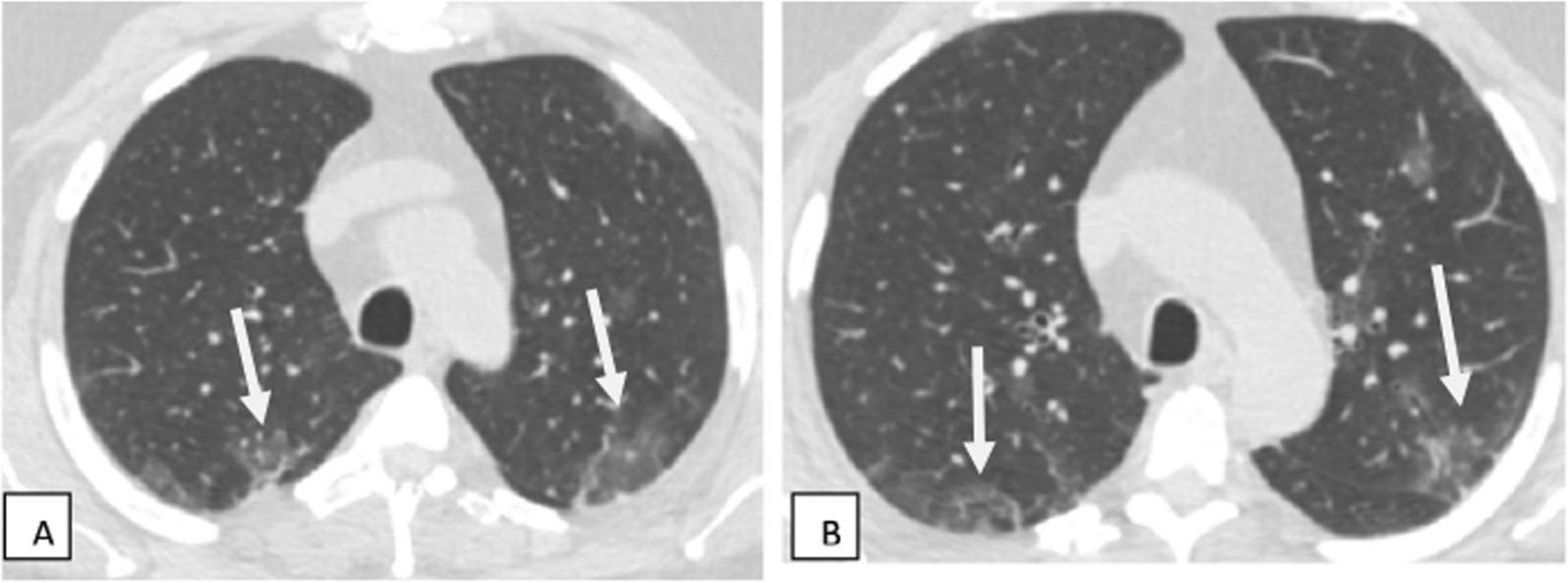
Male patient aged 62 years old with recent history of cough and mild dyspnea. Axial CT images (**A**, **B**) show ill-defined peripheral patchy areas of ground-glass densities seen at both lungs with subpleural position (white arrows) (mild severity)

**Fig. 2:**
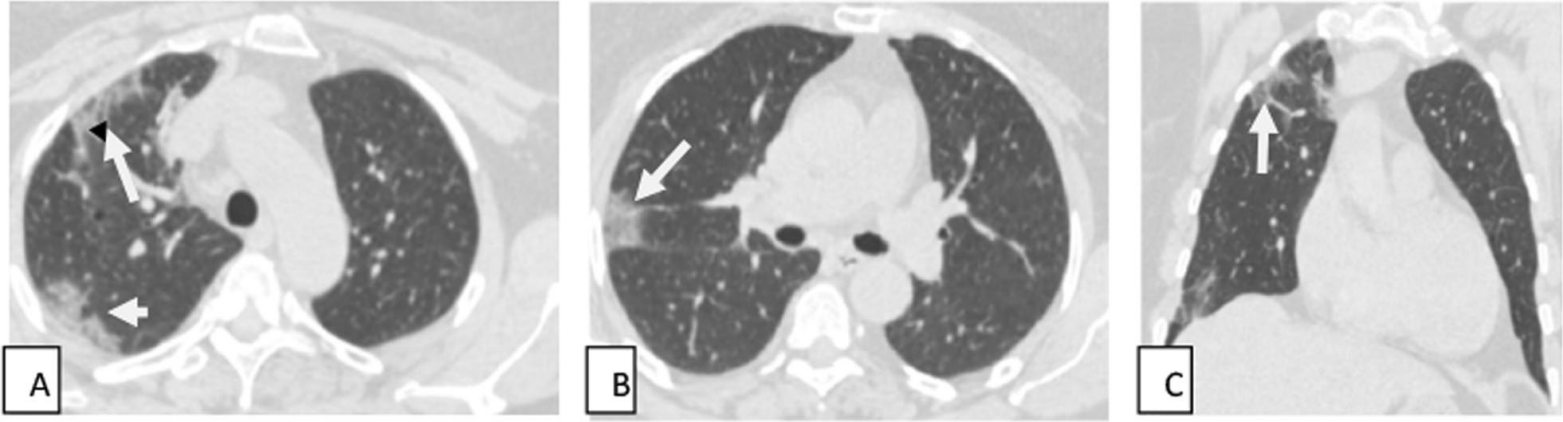
Male patient aged 56 years presented by loss of smell and taste with low-grade fever. Axial (**A**, **B**) and coronal (**C**) CT cuts show bilateral multifocal ground-glass opacities, and smaller areas of consolidation and areas of interstitial thickening giving crazy-paving appearance (white arrows) are seen involving both lung fields with peripheral/subpleural predominance (mild to moderate severity)

**Fig. 3:**
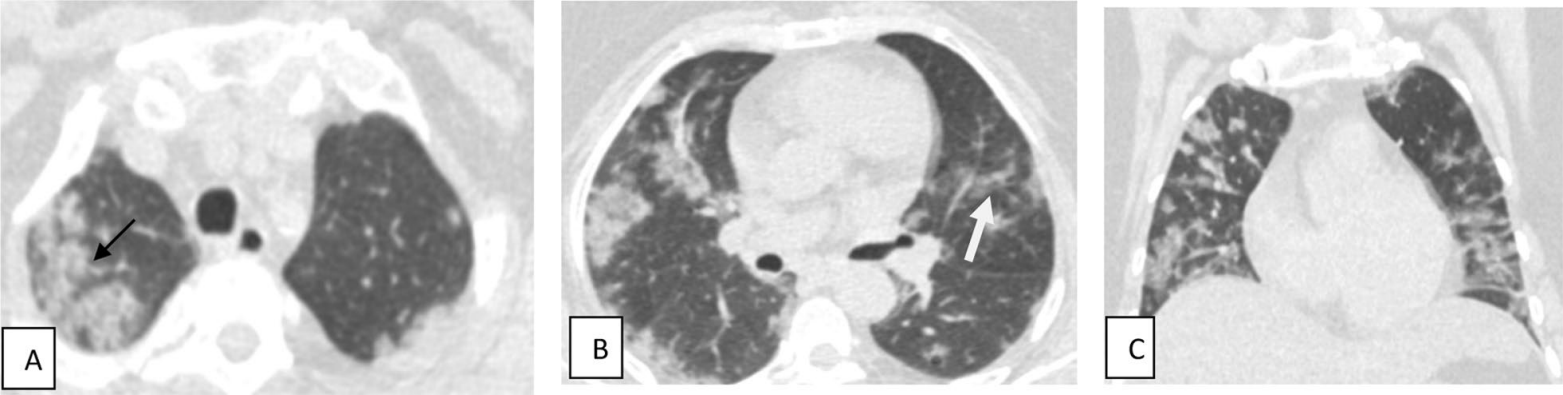
Female patient 46 years old presented by cough, dyspnea and fever. Axial (**A**, **B**) and coronal (**C**) CT images show bilateral multifocal ground-glass opacities (white arrow); areas of consolidation and areas of interstitial thickening giving crazy-paving appearance (black arrow) are seen involving both lung fields with peripheral/subpleural predominance (moderate to severe severity)

**Fig. 4:**
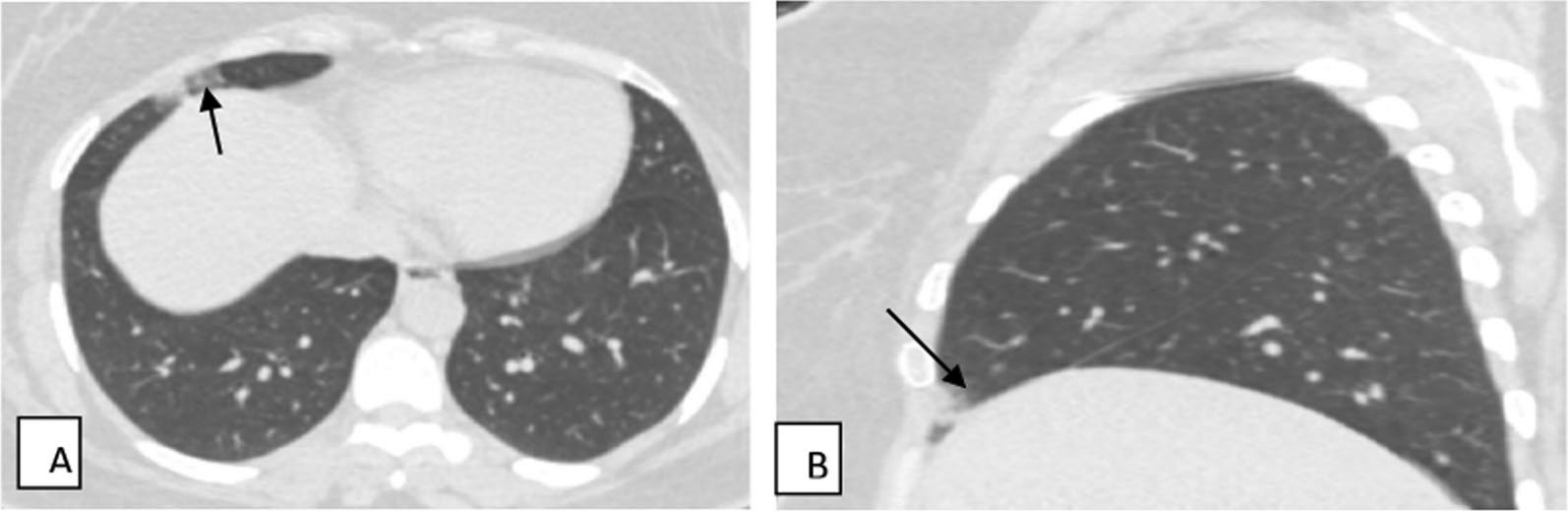
Female patient aged 34 years old presented by loss of smell and taste only. Axial (**A**) and coronal (**B**) CT cuts show single patchy area of ground-glass density is seen at right middle lobe (black arrow)

**Fig. 5:**
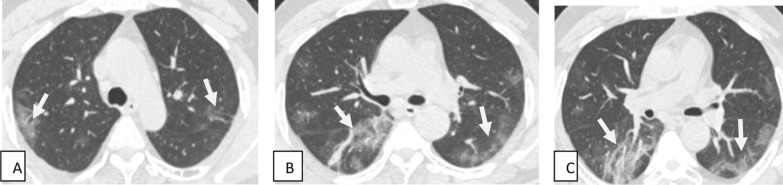
Male patient aged 54 years with cough, fever and myalgia. Axial (**A**–**C**) CT images show bilateral multifocal areas of ground-glass density (white arrows) seen at both lung fields with peripheral subpleural predominance (mild severity)

**Fig. 6:**
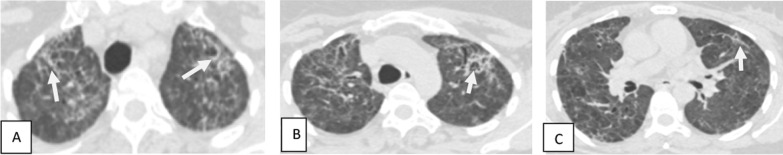
Female patient aged 56 years old, with history of COVID-19 infection 3 month ago, presented by dyspnea on exertion and tachypnea. Axial (**A**–**C**) CT cuts show bilateral diffuse interstitial thickening (white arrows) in the form of mild peri-bronchial cuffing and mild interlobular septal thickening with subpleural reticulation

**Fig. 7:**
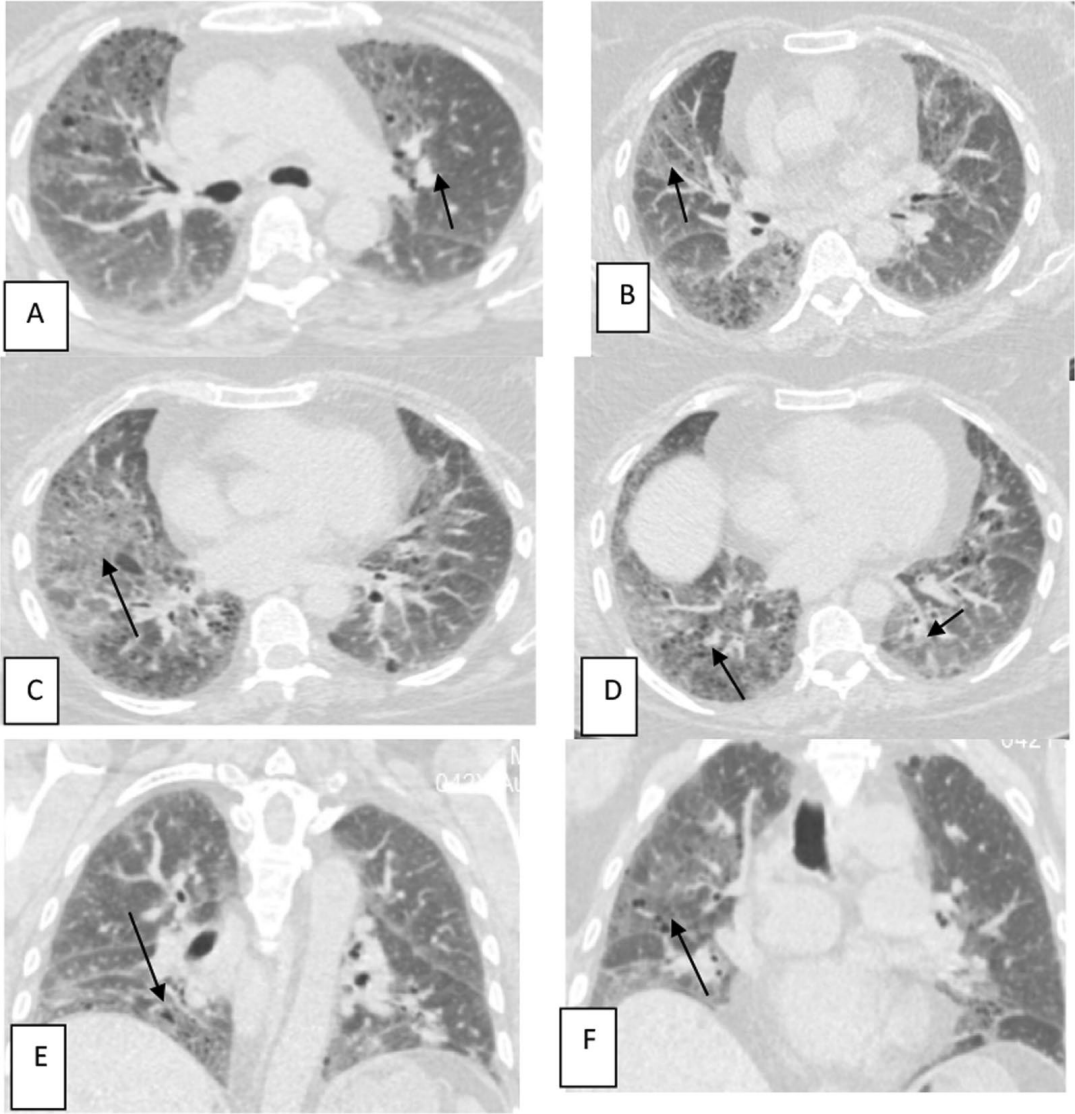
Female patient aged 35 years old, with history of COVID-19 infection 3 month ago, presented by dyspnea on exertion and tachypnea. Axial (**A**–**D**) and coronal (**E**, **F**) CT images show multiple patchy areas of ground-glass density with reticular infiltration and interlobular septal thickening, associated with multiple tiny cysts (< 1 cm) (black arrows); with lower lobes predominance, these changes are consistent with post-COVID-19 interstitial lung fibrosis

**Fig. 8:**
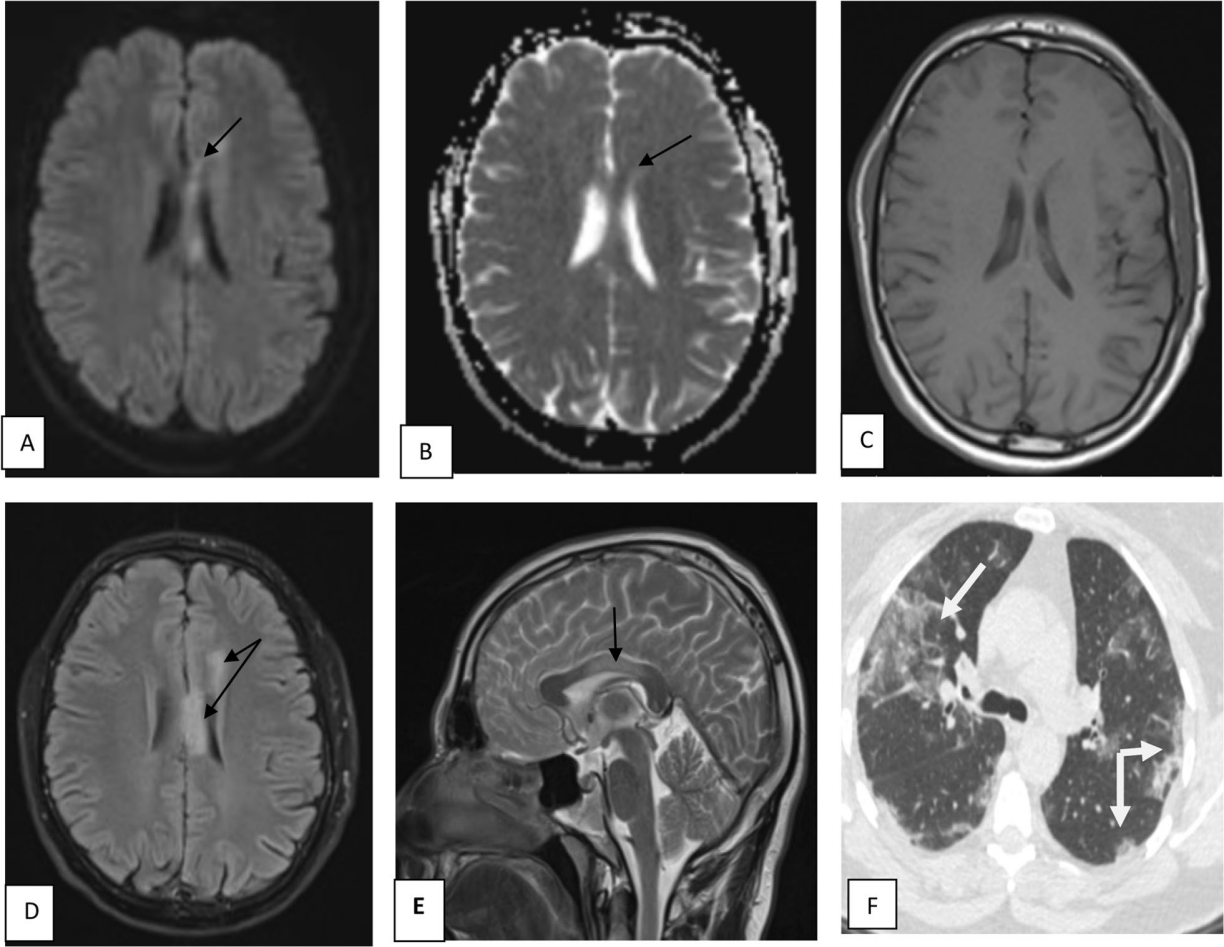
Male patient aged 29 years old, with history of COVID-19 infection 1 month ago, presented by headache, weakness in right side of the body and tingling started 3 days ago. MRI brain showed well-defined callosal and pericallosal left frontal area of altered signal intensity (black arrows) denoting subacute infarction, displaying high signal intensity in DWI (**A**) and intermediate signal intensity in ADC map (**B**) denoting free diffusion. The lesion displays isointense signal in T1WI (**C**) and high signal intensity in FLAIR sequence (**D**) and T2WI (**E**). Axial CT chest cut (**F**) of the same patient shows bilateral peripheral ground-glass opacities (white arrows) (moderate severity)

**Fig. 9:**
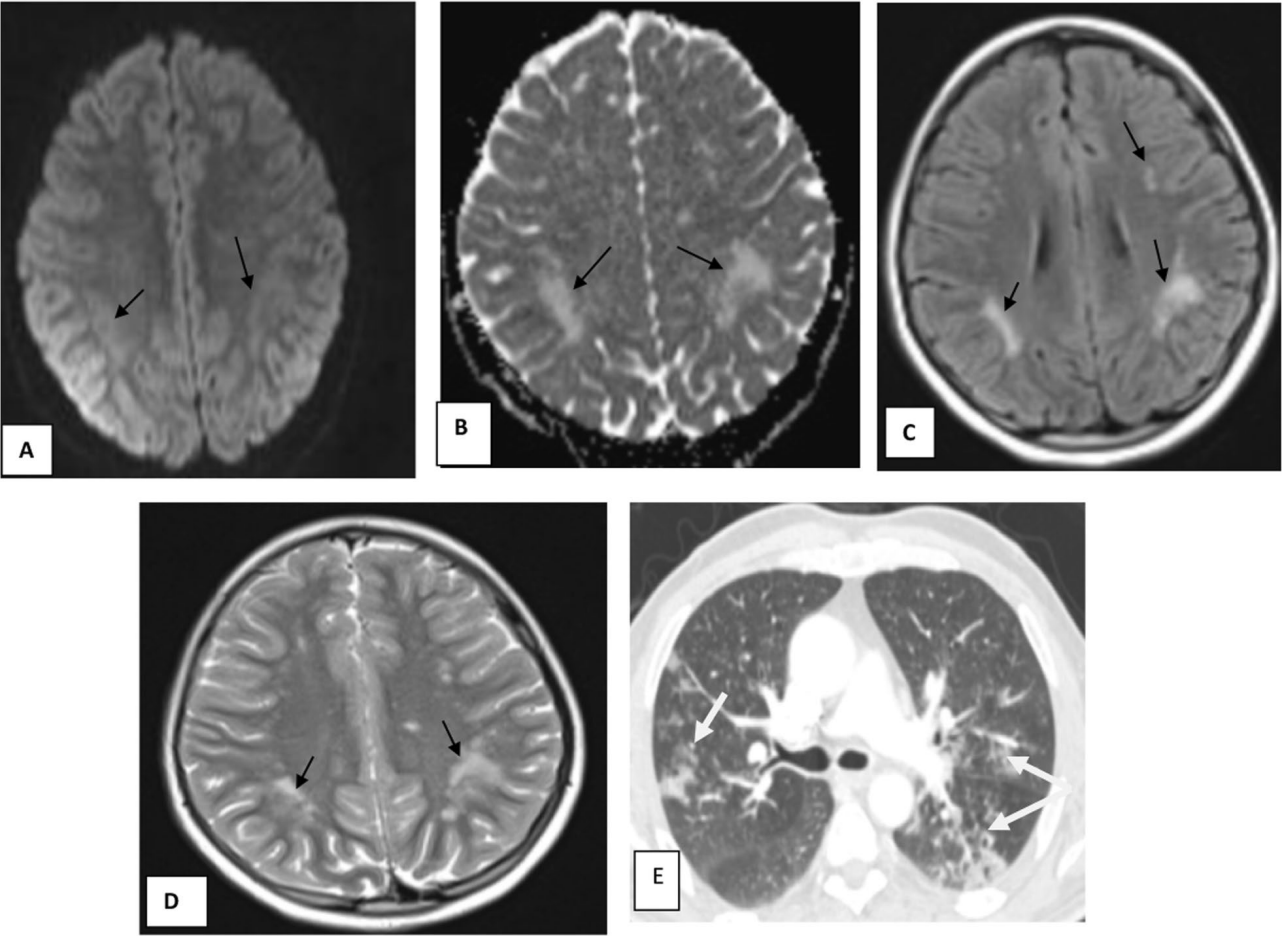
Male patient aged 28 years old, with history of COVID-19 infection. MRI of brain shows multiple patchy areas of abnormal signal intensity seen at subcortical white matter bilaterally (black arrows). They display high signal intensity in FLAIR and T2WI sequences (**C**, **D**), no diffusion restriction at DWI and ADC (**A**, **B**), no surrounding edema or mass effect. ADEM is considered in this case. Axial CT (**E**) chest cut of the same patient shows bilateral peripheral ground-glass opacities (white arrows) (moderate severity)

**Fig. 10:**
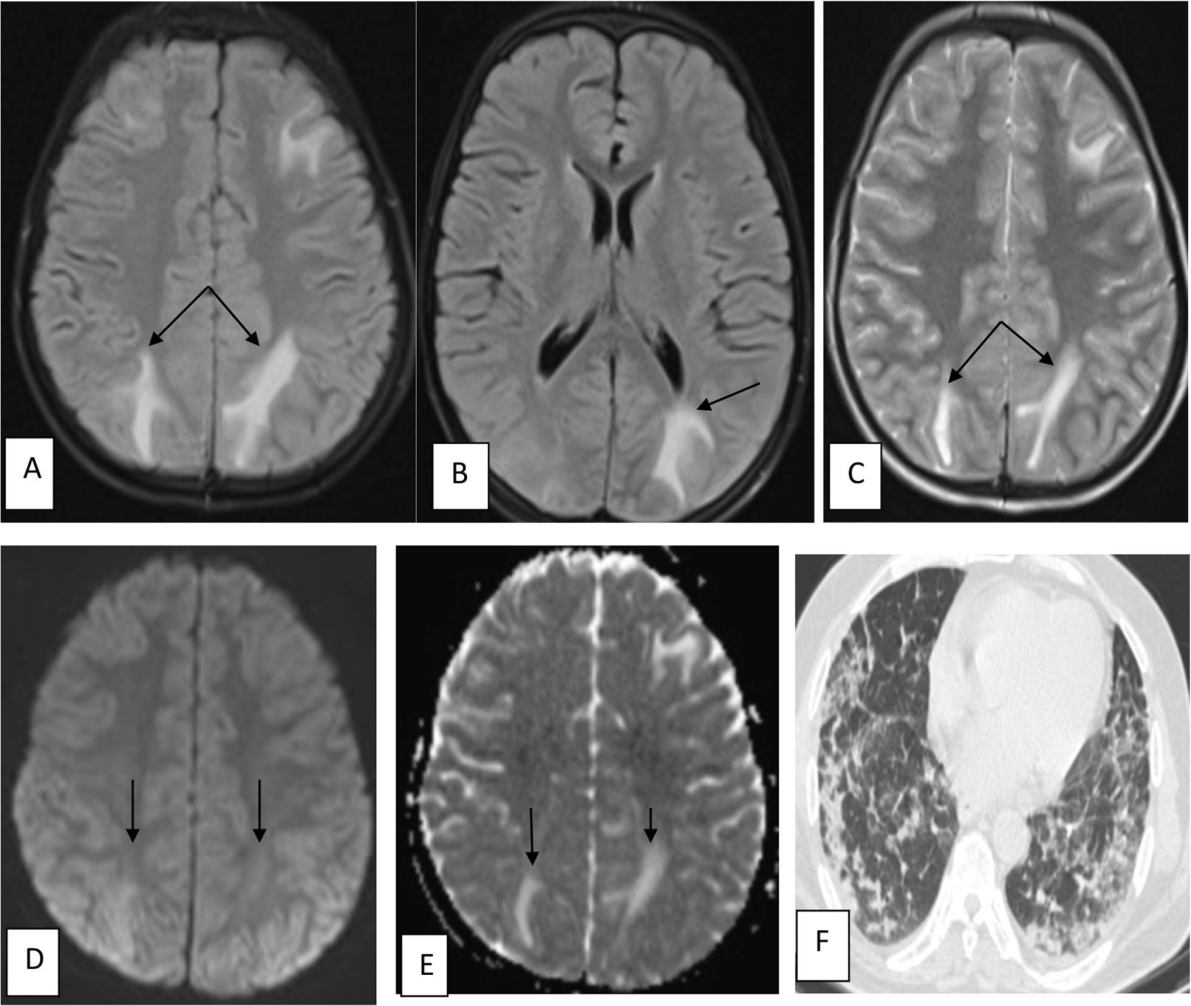
Female patient aged 40 years old known to be hypertensive with recent history of COVID-19 developed altered consciousness. MRI brain shows multiple nearly symmetrical subcortical patchy areas of altered signal intensity are seen at both high parietal and, occipital regions (black arrows) displaying high signal in FLAIR and T2 WI (**A**–**C**), free diffusion in DWI & ADC (**D**, **E**) this MRI features are suggestive of PRES (posterior reversible encephalopathy syndrome). Axial CT (**F**) chest cut of the same patient shows bilateral peripheral ground-glass opacities mixed with crazy-paving appearance (severe severity)

**Fig. 11:**
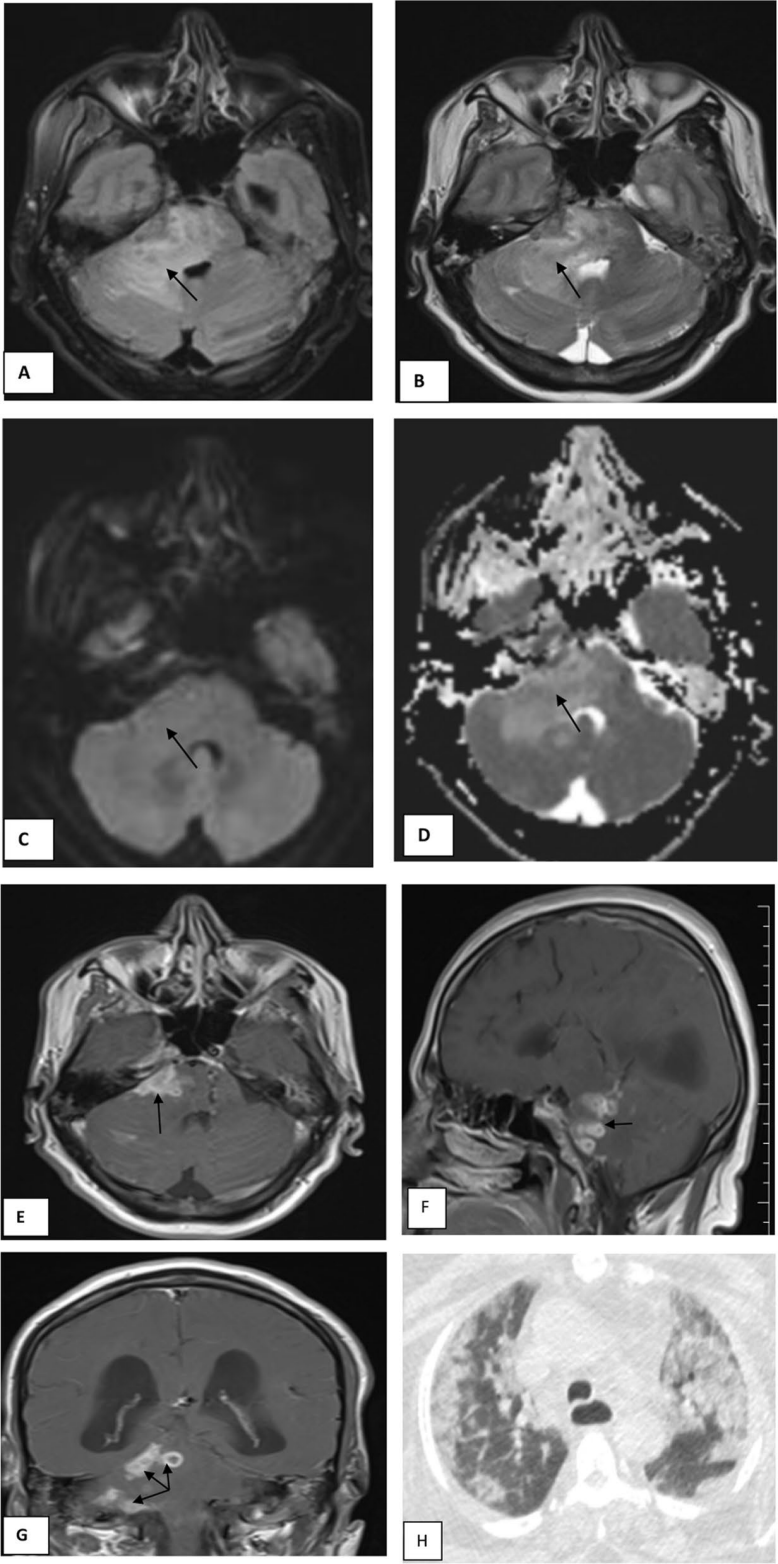
Male patient aged 48 years with recent history of COVID-19 since 3 weeks presented with fever, convulsions, altered consciousness and lethargy. FLAIR and T2WI sequences (**A**, **B**) show patchy area of high signal intensity seen at right mid brain, pons, right middle cerebellar peduncle as well as right cerebellar hemisphere. Diffusion images and ADC (**C**, **D**) show free diffusion (black arrows). Axial, sagittal and coronal (**E**–**G**) post-contrast T1 images show multiple ring enhancing lesions at fore-mentioned sites, this together with enhancing nodular meningeal thickening at right cerebello-pontine angle, right ambient cistern, right meckle’s cave and right cavernous sinus (black arrows) … this picture suggestive for post-COVID-19 meningoencephalitis. Axial CT (**F**) chest cut of the same patient shows bilateral peripheral ground-glass opacities mixed with crazy-paving appearance (severe severity)

The present study included 80 patients having recent or previous history of COVID-19 infection presented with respiratory and neurological symptoms. Demographic and associated comorbidities in the studied patients are recorded in Table 1. Most of COVID-19 patients came with respiratory symptoms and some with neurological manifestations as well. The most common patients’ complaints were rhinitis, persistent fever ranging from mild to severe fever, sore throat, dry cough, shortness of breath, fatigue, loss of taste and smell, seizers, headache, low mode and anxiety, stroke paresis and paralysis and altered mental status (Table 2). The reported CT findings of patients are present in Table 3 and Figs. 1, 2, 3, 4, 5, 6, 7. MRI done to 20 patients with neurological manifestation showed that 8/20 (40%) complaining of loss of taste and smell (5) and others (3) with severe headache and prolonged fatigue had no abnormalities. Twelve patients (60%) had abnormalities. MRI successfully detected serious COVID complications, namely infarction 7/12 (66.7%) {major 3″25″ and lacunar infarction 4″33.3″}, followed by acute disseminated encephalomyelitis (ADEM) 3 (25%), posterior reversible encephalopathy syndrome (PRES) 1/12 (8.3%) and meningoencephalitis 1 (8.3%). Correlation between CT and MRI of patients’ with respiratory and neurological manifestations is present in Table 4 (Figs. 8, 9, 10, 11).

**Table 1:** Demographic and associated comorbidities in studied patients

Characteristics	Number	Percent (%)
All patients	80	100
Age (years)		
Range	28–78	
Mean ± SD	57. 84 + 12.58	
Sex, no. (%) of patients		
Male	54	67.5
Female	26	32.5
BMI (Kg/m^2^) mean ± SD	30.4 ± 7.6	
Associated comorbidities		
Studied *n* (%)	80	100
No associated comorbidities	19	23.75
Hypertension	32	40
Cardiac	26	32.5
Pulmonary	30	37.5
Diabetes	40	50
Hepatic	10	12.5
COVID-19 severity		
Mild	24	30
Moderate	46	57.5
Severe	30	37.5

Multiple patients had multiple symptoms

*No* number, *%* percentage, *BMI* body mass index

**Table 2:** Symptoms at presentation and no. (%) of studied patients

Symptoms at presentation	Number	Percent (%)
Rhinitis	62	77.5
	24	30
Mild persistent fever	35	43.75
	55	68.75
Severe persistent fever	18	22.5
	8	10
Sore throat and dry cough	62	77.5
	60	75
Fatigue and poor appetite	14	17.5
Difficulty of breathing	28	35
Loss of taste and smell	32	40
Headache	16	20
Seizers	24	30
Low mode and anxiety	25	31.25
Stroke, paresis and paralysis	22	27.5
Altered mental status	8	10
Gait imbalance	80	100
Dizziness		
Myalgia		
Depression		
Total		

Multiple patients had multiple symptoms

**Table 3:** CT at presentation and no. (%) of studied patients

Characteristics	No. of patients	Percentage (%)
Normal lung shadows	12	15
Presence of GGOs alone	30	37.5
Presence of GGOs with consolidation	48	60
Presence of consolidation alone	12	15
Reticulation	6	7.5
Reticulation and GGOs (crazy paving)	11	13.75
		12.5
Fibrotic-like changes	10	100
Total studied	80	

Multiple patients had multiple lung findings

*No* number, *%* percentage

**Table 4:** Correlation between MRI findings and CT severity index

MRI findings	CT chest findings			
	Mild	Moderate	Severe	Total
	≤ 10	11–20	≥ 20	
Normal	5	3	0	8
Major infarction	0	2	1	3
Lacunar infarction	1	1	2	4
ADEM	0	1	2	3
PRES	0	0	1	1
Meningoencephalitis	0	0	1	1
Total	6	7	7	20

*ADEM* acute disseminated encephalomyelitis, *PRES* posterior reversible encephalopathy syndrome

## Discussion

COVID-19 is optimized to disseminate rapidly and widely [14], primarily through the respiratory tract by droplets, respiratory secretions and direct contact [15]. Therefore, chest CT had to be performed at sites with less traffic to avoid exposure of other patients and staff.

Chest CT is an important and fast imaging tool for the diagnostic workup of infected patients. Chest CT scan can predict the severity of the disease by showing the percentage of lung involvement and thus give an idea about the prognosis of the disease. Therefore, an appropriate and optimal management would be overtaken earlier, thus decreasing patient hospitalization and mortality rate. The prevalence of chest CT abnormalities in COVID-19 is dependent on the stage and severity of the disease [16].

The current study showed that COVID-19 infection affects old age males more than females, and this could be to the biological differences between men and women [17]. The old age may be attributed to the coexisting morbidities in those patients and other factors relating to aging. This agreed with results by Liu et al. [18].

Normal chest CT findings in the present study were detected in 12 (15%) in symptomatic patients and this could be attributed to early time of examination as previously reported by Adams et al. [19]. However, Wang et al. [20] found a nonnegligible number of symptomatic cases with normal chest CT findings during the later stage of the infection.

The ground-glass opacities (GGOs) whether alone 30 (37.5%) or with consolidation were the most common pattern of pulmonary changes in COVID-19 patients and were seen in 48 out of 80 (60%) patients. This coincided with previous other studies, Parry et al. [21], Omar et al. in Egypt [17] and Adnan et al. [22], who showed that ground-glass pattern was the most common CT pattern in their studies.

Consolidation alone was found in 12 (15%) in this study; this is in comparison to the findings reported by Omer et al. [17] and Adnan et al. [22] who recorded consolidation in 23% and 9% in their studies respectively. This variation could be attributed to the timing at which CT examination is performed as consolidation with or without GGO is seen in the 2nd and 3rd weeks of infection course.

CT in the present study showed singular or multiple irregular areas of GGO or consolidation or both in 60 of the 80 (65%) patients. This was similar to previous studies of Li and Xia [23], who detected GGOs or consolidation or both in 49 of the 51 (96.1%) patients.

In the remaining patients, reticular pattern appears in 6 (7.5%) as a complex network of linear opacities on CT images, which is caused by interlobular and intralobular septal thickening as a result of lymphocytes infiltration as previously reported [24]. Previous studies reported that the appearance of a reticular pattern with interlobular septal thickening is one of the most common CT findings in patients with COVID-19 [25]. The prevalence and frequency of reticular patterns may indicate increase in COVID-19 disease progression as suggested by previous researchers [26].

Some CT images of COVID-19 patients 10 (12.5%) showed the feature of fibrosis; in the shape of reticulations, bronchiectatic changes or even honeycomb patterns. However, appearance of fibrosis may indicate either that the pulmonary lesions have been absorbed or signify fibrous hyperplasia.

Fibrotic changes are progressive and may result in irreversible interstitial lung disease, which may lead to the decline of pulmonary function, worsening of symptoms, poor quality of life and early mortality as previously reported by researchers [27].

The COVID-19 outbreak primarily targets the respiratory epithelium but also has neuro-invasive potential. Indeed, neuropsychiatric manifestations, such as fatigue headache, dizziness and delirium, are consistently observed in COVID-19 [28].

In the present study, headache was the most common central CNS manifestation, while smell and taste impairment were the commonest manifestations affecting the peripheral nervous system (PNS). These findings are in agreement with the study of Agarwal et al. [29].

Brain MRI is a feasible and important imaging modality in selected patients with COVID-19 pneumonia. MRI findings of the present study showed that eight cases out of 20 patients with neurological manifestation having mild or moderate coronavirus infection were normal. Our results coincided with Paterson et al. [30], who showed that MRI results or cerebrospinal fluid findings might be normal or suggestive of encephalitis for patients with CNS symptoms.

In the current, study 12 patients with coronavirus infection showed infarction in 7 cases (major or lacunar). The rest of cases (five) with moderate or severe acute infection had the picture of ADEM in 3 cases, one case had PRES and one case had meningoencephalitis. Such findings were similar to previous studies that showed the correlation between severity of COVID-19 infection and MRI findings of brain. Mao et al. [8] showed that mild cases of COVID-19 present with headache or dizziness, whereas those with severe disease can also develop an encephalopathy with agitation and signs of corticospinal tract dysfunction. Paterson et al. [30] found acute COVID-19 inflammatory CNS syndromes encephalitis, acute disseminated encephalomyelitis (ADEM), myelitis and ischemic strokes. Nepal et al. [31] found that respiratory syndrome coronavirus may damage the central nervous system (CNS).

Multiple recent reports suggest that COVID-19 is associated with acute cerebrovascular disease, including intracranial hemorrhage, large-vessel occlusion, acute ischemic stroke and dural venous sinus thrombosis [32].

Thus, the presence of neurological complications could be a bad prognostic element in COVID-19 patients. In the present study, ADEM, PRES and meningoencephalitis were detected only in moderate or severe COVID-19 cases. Our results coincided with Khatoon et al. [33], who found that neurological involvement in COVID-19 patients carries a bad prognostic indication. It was associated with more frequent need of mechanical ventilation and higher risk of mortality. In addition, Gusev et al. [34] found a relationship between the severity of COVID-19 and the severity and frequency of neurological manifestations.

Further studies needed on a large number of patients to clarify the value of chest CT for prognostication in COVID-19 and the pathogenesis and long-term prognosis of CNS involvement in patients with COVID-19, including correlation with patient outcome.

## Conclusion

Lung manifestations are common in COVID-19 patients than neurological manifestations. The presence of fibrotic changes in the lung could predict severe COVID-19 affection and bad prognosis. There might be an association between neurological manifestations and poor outcome in COVID-19 patients.

## Data Availability

All data and materials are available.

## Abbreviations

COVID-19: Coronavirus disease 2019
CT: Computed tomography
ICU: Intensive care unit
MRI: Magnetic resonance imaging
GGOs: Ground-glass opacifications
ADEM: Acute disseminated encephalomyelitis
PRES: Posterior reversible encephalopathy syndrome
CSF: Cerebrospinal fluid
SD: Standard deviation
CNS: Central nervous system
